# Does Oxygen Content Play a Role in Spontaneous Closure of Perimembranous Ventricular Septal Defects?

**DOI:** 10.3390/children8100881

**Published:** 2021-10-02

**Authors:** Pier Paolo Bassareo, Giuseppe Calcaterra, Martino Deidda, Andrea Raffaele Marras, Giuseppe Mercuro

**Affiliations:** 1 School of Medicine, Mater Misericordiae University Hospital and Our Lady’s Children’s Hospital Crumlin, University College of Dublin, D07 R2WY Dublin, Ireland; 2 Postgraduate Medical School, University of Palermo, 90127 Palermo, Italy; peppinocal7@gmail.com; 3 Department of Medical Sciences and Public Health, University of Cagliari, 09042 Cagliari, Italy; martino.deidda@icloud.com (M.D.); alalazo@gmail.com (G.M.); 4 Department of Pediatrics and Surgical Sciences, University of Cagliari, 09042 Cagliari, Italy; silviamarras3@gmail.com

**Keywords:** ventricular septal defect, congenital heart disease, haemoglobin, anaemia, iron

## Abstract

(1) Background: the impact of a series of laboratory parameters (haemoglobin, haematocrit, foetal haemoglobin, peripheral oxygen saturation, iron, transferrin, ferritin, and albumin) on perimembranous ventricular septal defects spontaneous healing was tested. (2) Methods: one hundred and seven patients were enrolled in the study (57% males; mean age 2.1 ± 0.4 years) and were subsequently subdivided into two groups: self-healing (*n* = 36) and in need of intervention (*n* = 71). Self-healing subjects were defined on the basis of an absence of residual shunts at colorDoppler across the previous defect. (3) Results: no statistically significant differences were reported in the size of perimembranous ventricular septal defects between the two groups (*p* = ns). Conversely, prevalence of anaemia was significantly higher in those requiring intervention than in the self-healing group (*p* < 0.03), while haemoglobin, iron, ferritin, and albumin levels were lower (*p* < 0.001, *p* < 0.05, *p* < 0.02, *p* < 0.007, respectively). In multivariable linear regression analysis, only haemoglobin and albumin were found to be associated with spontaneous closure (*p* < 0.005 and *p* < 0.02, respectively). In multiple logistic regression analysis, haemoglobin independently increased the probability of self-healing of perimembranous ventricular septal defects (*p* = 0.03). All patients needing an interventional closure of perimembranous ventricular septal defects presented with haemoglobin <12.7 g/dL. (4) Conclusion: the self-resolution of perimembranous ventricular septal defects seems to rely on numerous factors, including oxygen content, which is likely to promote cell proliferation as well as tissue regeneration. Haemoglobin blood concentration seems to influence the natural history of perimembranous ventricular septal defects and improvement of anaemia by supplementation of iron intake might represent a simple and reliable method to promote self-healing.

## 1. Introduction

**Figure 1:**
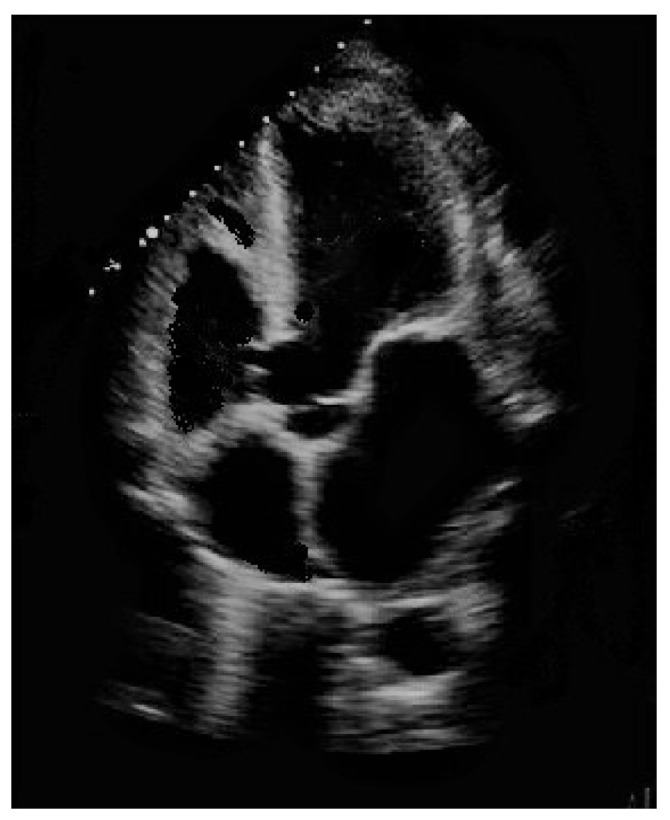
Perimembranous ventricular septal defect partially occluded by tricuspid valve accessory tissue.

Ventricular septal defect, manifested either as an isolated event or in conjunction with other cardiac abnormalities in syndromic and non-syndromic patients, is by far the most frequently encountered congenital heart defect (CHD) after bicuspid aortic valve in clinical practice, accounting for approximately 20% of all diagnoses when isolated [1,2]. Numerous classifications for ventricular septal defects have been proposed, although it is indisputable that perimembranous ventricular septal defect is the most frequently observed subtype in children (Figure 1), whilst a muscular presentation is most common in newborns. 

At birth, approx. 8% of detected ventricular septal defects are perimembranous, i.e., involving both the membranous septum and the adjacent muscular area [3]. Diagnosis of ventricular septal defect is usually simple, being mainly based on echocardiography, a widely used technique that also facilitates follow-up of the course of ventricular septal defect [4].

Numerous studies have demonstrated self-healing of perimembranous ventricular septal defects in 10–30% of cases, thus avoiding the potential peri-procedural complications associated with repairs performed by means of conventional surgery or transcatheter-occluding devices [5,6].

Anaemia is frequently observed in both children and adults affected by all types of CHD, including ventriular septal defects at all ages [7,8]. Renal impairment, abnormal iron metabolism, malnutrition, and circulatory congestion contribute independently toward the occurrence of anemia in CHD [9].

This study aimed to assess the impact of a series of laboratory parameters on spontaneous full healing of perimembranous ventricular septal defect: haemoglobin, haematocrit, foetal haemoglobin, peripheral oxygen saturation, iron, transferrin, ferritin, and albumin blood levels.

## 2. Materials and Methods

### 2.1. Patients in the Study

In this retrospective study, medical records of patients affected by perimembranous ventricular septal defect attending the Paediatric Cardiology Unit of the University of Cagliari (Italy) from January 1986 to February 2006 were examined. No additional subjects could be evaluated due to closure of the above-cited unit. Following a review of hardcopy medical records and electronic health records, one hundred and seven patients were included in the study (57% males), ranging from 1 day of life to 6 years (mean age 2.1 ± 0.4 years). Clinical and echocardiographic characteristics of study participants are listed in Table 1.

Inclusion criteria were: presence of a perimembranous ventricular septal defect without other CHD, with the exception of transient patent ductus arteriosus, small atrial septal defect and/or one or more minor muscular ventricular septal defects; when surgical closure was indicated due to a large volume left-to-right shunt (Qp/Qs > 2:1), in the presence of significant pulmonary arterial hypertension (pulmonary arterial pressure > 50% systemic), clinical signs of congestive heart failure, banding of the pulmonary artery, or when closure of the perimembranous ventricular septal defect was deemed clinically necessary by the treating physician. Exclusion criteria comprised: ventricular septal defect exceeding 10 mm and/or a ventricular septal defect size/aortic diameter ratio higher than 2/3 (in which case spontaneous closure is improbable); patients presenting with multiple severe muscular ventricular septal defects and/or complex CHD; presence of ongoing bacterial infections/sepsis; haematological disorders (mainly haemolytic disease of the newborn); insufficient laboratory findings. Patients were subdivided into two groups: self-healing (*n* = 36) and those in need of intervention (*n* = 71).

Diagnosis was formulated and echocardiographic follow-up implemented. A HP/Philips Sonos 5500, (Amstedam, The Netherlands) echo machine coupled with two probes (3–5 Mhz and 8–10 Mhz) was used. Patients diagnosed with perimembranous ventricular septal defect were followed-up until either the hole in the interventricular septum closed spontaneously or surgical closure was performed. The net defect was measured, which may be smaller than the true due to tricuspid pouch formation. The self-healing group was defined on the basis of an absence of residual shunts at colorDoppler across the former perimembranous ventricular septal defect.

Written informed consent was waived owing to the retrospective nature of the research, as per Italian Law. The research was formally approved by the internal Ethics Committee of the University of Cagliari (PG/2015/1859) and conducted in accordance with the Helsinki declaration.

### 2.2. Laboratory Tests

The following blood parameters capable of affecting tissue oxygenation were evaluated on a blood sample taken from an antecubital vein-haemoglobin: normal haemoglobin values for children vary according to the child’s age, sex, and race. Anaemia is defined in the presence of a haemoglobin value at or below the 2.5th percentile for age, race, and sex [9,10].

- haematocrit [11,12]
- foetal haemoglobin: the most important oxygen-transport protein in the human foetus [13]
- oxygen saturation [14]
- serum iron levels [15].
- transferrin [16]
- ferritin [16]
- albumin [17,18] (See Table 2, Table 3 and Table 4).

### 2.3. Statistical Analysis

The results obtained in the study population (*n* = 107) were analysed and the findings obtained in the self-healing group subsequently compared with those from the group requiring intervention using the parametric Student’s t-test, as the sample was normally distributed. Normality was assessed by means of the Kolmogorov–Smirnov test. Multivariate analysis was also performed to analyse more than one outcome variable. The model included the hematic variables which tested statistically significantly different between the two subgroups (i.e., haemoglobin, iron, ferritin, albumin) along with age and gender. For the purpose of this paper, statistical significance was set at <0.05. Commercially available computer software (SPSS version 22.0, SPSS Inc., Chicago, IL, USA) was used for all analyses.

## 3. Results

The findings obtained in the self-healing group vs the group requiring intervention are summarized in Table 5.

Regarding the time required by the two groups to reach their outcome, it was statistically significant. Since no statistically significant differences were detected in the dimensions of ventricular septal defect between the two groups, the need for an early intervention was likely due to a drop in pulmonary vascular resistances, which led to a significant left-to-right shunt and in turn to pulmonary overflow and heart failure. Furthermore, anaemia was significantly more prevalent amongst subjects requiring intervention than in the self-healing group (*p* < 0.03), while haemoglobin, iron, ferritin, and albumin levels were lower (*p* < 0.001, *p* < 0.05, *p* < 0.02, *p* < 0.007, respectively).

At multivariable linear regression analysis only haemoglobin and albumin featuring an association with spontaneous closure of perimembranous ventricular septal defect (*p* < 0.005 and *p* < 0.02, respectively). Multiple logistic regression analysis revealed that haemoglobin independently raised the probability that self-resolution of ventricular septal defect would be achieved (*p* = 0.03).

**Figure 2:**
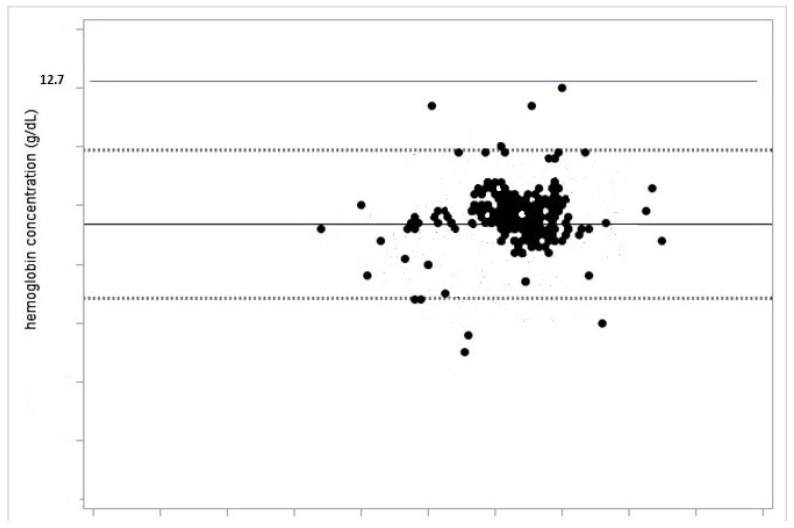
All the patients in the study whose perimembranous ventricular septal defect did not heal by itself had a haemoglobin level less than 12.7 g/dL.

Haemoglobin levels in all patients in the needing intervention group was less than 12.7 g/dL (Figure 2). 

## 4. Discussion

In clinical practice, the most commonly observed CHD is represented by ventricular septal defect, of which a considerable percentage displays a tendency to decrease in size and to self-heal. Numerous mechanisms of spontaneous closure of perimembranous ventricular septal defect have been described previously [19].

The present study excluded both muscular ventricular septal defects and other subtypes. The former present with a completely different natural history compared with the perimembranous form, i.e., spontaneous healing in 85%–90% of cases due to a progressive muscularization of the left ventricle, while for other subtypes the rate of spontaneous healing is very low [20].

Furthermore, perimembranous ventricular septal defects with dimensions exceeding 10 mm and/or a ventricular septal defect size/aortic diameter >2/3 ratio were excluded from the study due to the improbability of spontaneous closure occurring [21]. This might explain why our findings highlighted no significant differences in ventricular septal defect size between the two groups studied.

A slight prevalence of the disease amongst males was detected in our cohort. Generally speaking, no significant sex-related differences in ventricular septal defect prevalence are reported in literature [22].

Numerous anatomical parameters have been put forward as potential independent predictors influencing the spontaneous healing of ventricular septal defects. However, more detailed knowledge should be acquired, and the biological mechanisms implicated better elucidated [23]. Accordingly, a series of hematic factors potentially underlying the spontaneous resolution of perimembranous ventricular septal defects were examined.

Anemic infants have ventricular septal defects more frequently than those with normal haemoglobin levels [24]. Haemoglobin is capable of promoting tissue regeneration, cells proliferation and wound healing, as both processes rely heavily on oxygenation. There is no doubt that an adequate supply of nutrients and oxygen to regenerating cells is crucial for their survival and functional maintenance [25].The natural healing of perimembranous ventricular septal defects involves a series of different mechanisms, including tissue growth from the remnant membranous septum or tricuspid valve and adhesion of tricuspid valve leaflets [19]. Tissue regeneration seems to be influenced by haemoglobin-related tissue oxygenation in a number of clinical scenarios [26]. Moreover, correct blood viscosity represents another factor of importance in promoting the adhesion of tricuspid valve leaflet to the tissue surrounding perimembranous ventricular septal defects [19]. Blood viscosity is intricately linked to haemoglobin content: the higher the haemoglobin levels, the higher the hematic viscosity and vice versa [27]. Anaemia, and subsequently reduced blood viscosity, may exert a negative influence on the above-stated adhesion, thus impinging on the natural resolution of ventricular septal defect [28]. Not only, but left-to-right shunt across ventricular septal defect may lead to a volume overload to the lungs, as expressed by QP/Qs ratio higher than 1.1, with consequent further dilution of haemoglobin [29]. Major and minor forms of thalassemia and other types of anaemia are considerably diffuse in Sardinia, i.e., the Italian region where the research was carried out, which may have negatively affected the number of self-healing patients in our cohort [30]. Our research showed a significantly lower prevalence of anaemia in the self-healing group than in those needing intervention. Our findings also suggest a value of 12.7 g/dL in haemoglobin content, above which level the self-healing of ventricular septal defect is unlikely. Overall, as confirmed also at multivariate analysis, haemoglobin blood concentration seems to influence the natural history of ventricular septal defect, and improvement of anaemia through supplementation of iron intake might represent a simple and reliable method of promoting the spontaneous healing of perimembranous vetricular septal defects [31].

Albumin levels were significantly higher in the self-healing group than in patients requiring intervention. In a previous study, hypoalbuminemia, commonly observed in patients with CHD, was found to be associated with an increased risk of death, even after adjustment for disease complexity, functional class, and other risk factors [32]. Low albumin levels are likely caused by acute and/or chronic heart disease due to systolic or diastolic left ventricular dysfunction, which are not uncommon in ventricular septal defect patients [33,34]. Some animal models have also suggested a role of albumin in promoting early wound healing [35,36].

This study is undoubtedly hampered by several limitations: (1) the study was of a single centre retrospective design and therefore contingent on the inherent bias associated with this type of study (missing data; referral and selection bias); (2) over the period examined (1986–2006), the majority of surgical procedures were delayed compared with current standard decision making and a smaller number of interventional perimembranous ventricular septal defect closures were performed [37]. This may have slightly influenced our findings; (3) other unevaluated factors of an epigenetic nature might have influenced the results [38]. In this setting, artificial intelligence and machine learning may represent a potential way to detect important predictors of perimembranous ventricular septal defect self-resolution. The superior performance of machine learning in detecting haemoglobin-related and genetic predictors of cardiovascular endpoints has been already shown in some papers [39,40]; (4) anaemia, or conversely, polycythaemia and ventricular septal defect are not uncommon in Trisomy 21 patients, although the possible effect of Down syndrome itself on self-healing was not investigated [41,42]; recently, a scoring system aimed at predicting spontaneous healing of perimembranous ventricular septal defect, mainly based on anatomical factors, was proposed, although it was not tested in this study [43].

## 5. Conclusions

Overall, the self-resolution of perimembranous ventricular septal defect seems to rely on numerous different factors, including oxygen content, which likely promotes cell proliferation as well as tissue regeneration. Accordingly, in the presence of low oxygen saturation at a high altitude, ventricular septal defects are more likely to remain open [44]. Furthermore, more in-depth studies are needed to better clarify this intriguing finding [45].

## Figures and Tables

**Table 1:** Characteristics of the patients in the study (mean ± standard deviation).

Age at study (years)	2.1 ± 0.4
Age at spontaneous closure (years)	1.6 ± 0.8
Age at intervention (years)	0.8 ± 0.1
Perimembranous VSD size (mm)	0.55 ± 0.18
VSD spontaneous closure	reduplication of trispid valve tissue (61%)
	adhesion of tricuspid valve leaflets (23%)
	prolapse of an aortic valve cusp (7.7%)
Number of surgeries in NIG	(56/71; 78.9%)
Percutaneous closures in NIG	(15/71; 21.1%)

VSD: ventricular septal defect; SHG: self-healing group; NIG: needing intervention group.

**Table 2:** Laboratory parameters tested in the study.

1. **Haemoglobin**: an iron-containing oxygen carrier protein present in red blood cells. As a general rule, oxygen is transported in the blood in two forms: dissolved in plasma and red blood cell water (around 2%) and reversibly bound to haemoglobin (about 98%). Haemoglobin may be saturated with no more than four oxygen molecules (oxyhaemoglobin) or desaturated without oxygen molecules (deoxyhaemoglobin). Its affinity for oxygen may impair or enhance the release of oxygen to tissues.	


2. **Hematocrit:** the fractional volume of blood occupied by red blood cells with levels depending on the age and, following adolescence, sex of the individual (Table 3). As a general rule, a rise in haematocrit increases oxygen concentration in the arteries and delivery to tissues. However, the latter may decrease in the presence of haemoconcentration and polycythaemia as a result of decreased venous return and cardiac output, respectively. However, as a compensatory mechanism, low flow velocity leads to extended transit time of red cells through the capillary network, thus facilitating preservation of tissue oxygenation. On the contrary, even in the event of a decrease in oxygen content caused by haemodilution (low haematocrit and normal blood volume), opposite mechanisms may contribute towards preserving tissue oxygenation by means of increased cardiac output and blood flow to the organs based on lower blood viscosity, reduced total peripheral resistance, and increased venous return.	data
	data
3. **Foetal haemoglobin:** at birth, 50%–95% of haemoglobin in an infant born at term is represented by foetal haemoglobin, although levels decline rapidly over the initial six months of life as synthesis of adult-type haemoglobin is activated and synthesis of foetal haemoglobin comes to a standstill. However, foetal haemoglobin has been detected in the blood of adults (<1% of all haemoglobin). The most striking difference compared with adult haemoglobin is represented by the observation that foetal haemoglobin displays a higher affinity in binding to oxygen than the adult form, thus facilitating the capture of oxygen deriving from the mother’s bloodstream by the foetus. A series of genetic abnormalities may induce a failure in the switch to adult haemoglobin synthesis, thereby heralding onset of a state of hereditary persistence of foetal haemoglobin into adulthood. This condition is usually asymptomatic and may at times alleviate the severity of certain haemoglobinopathies and thalassemias, which are not uncommon in Sardinia, i.e., the Italian region where the research was carried out.	data
	data
	data
	data
4. **Oxygen saturation:** the amount of oxygen travelling through the body with red blood cells. In humans, normal levels of oxygen saturation range from 95%–100%, with levels below 90% being considered low and resulting in hypoxemia. Blood oxygen levels of less than 80% (cyanosis) may affect organ development and function and should be promptly addressed. Oxygen saturation is usually measured by means of pulse oximetry. 5. **Serum iron levels:** laboratory test measuring the quantity of transferrin- and serum ferritin-bound iron (approx. 90% and 10%, respectively) present in blood. Approximately 65% of total body iron is bound to haemoglobin molecules, as part of a heme group, in red blood cells, with approx. 5% present in myoglobin molecules. Almost 30% of total body iron is stored mainly in the liver, bone marrow, and spleen as ferritin or hemosiderin. A lack of iron may result in onset of anaemia. 6. **Transferrin:** a group of blood plasma glycoproteins which binds to iron and regulates free iron levels in biological fluids. Transferrin synthesis occurs mainly in the liver. Increased plasma transferrin levels are frequently observed in patients affected by iron deficiency anaemia. As plasma transferrin level increase, a concomitant decrease in the percentage of transferrin iron saturation is manifested. 7. **Ferritin:** an intracellular protein responsible for the storage and controlled release of iron. Liver stores of ferritin represent the primary body reserve of iron and protect against iron deficiency. In the presence of low ferritin levels, iron deficiency may ensue, potentially resulting in anaemia. Low serum ferritin is a highly specific laboratory test used to detect iron-deficiency anaemia.	data
	data

**Table 3:** Haemoglobin hematic concentration (g/dL) normal values.

Newborn	15.0–21.0
Baby (3–6 months)	9.5–12.5
Child (1–18 years)	11.0–13.0
Caucasian Adult female	11.5–15.5
Caucasian Adult male	13.5–17.5

**Table 4:** Haematocrit normal values (%).

Newborns	55–68%
One week of age	47–65%
One month of age	37–49%
Three months of age	30–36%
One year of age	29–41%
Ten years of age	36–40%
Adult males	42–54%
Adult women	38–46%

The above blood parameters were examined every 3–6 months until ventricular septal defect self-healing was achieved or surgical/interventional procedures were under taken and their averaged value used for statistical analysis.

**Table 5:** Results (SHG vs NIG).

	SHG (*n* = 36)	NIG (*n* = 71)	*p* Value
Outcome (years)	1.6 ± 0.8	0.8 ± 0.1	0.0001
VSD dimensions (mm)	0.54 ± 0.2	0.56 ± 0.1	ns
Prevalence of anemia (%)	8.8	14.7	<0.03
Gender (males %)	58.2%	55.8%	ns
BSA (m^2^)	0.553 ± 0.028	0.561 ± 0.025	ns
Hb (g/dL)	13.5 ± 0.2	11.6 ± 0.1	<0.001
Ht (%)	43.3 ± 0.4	42.9 ± 0.3	ns
HbF (%)	0.9	0.8	ns
SaO_2_ (%)	98%	97%	ns
Iron (µg/dL)	118.3 ± 7.5	115.1 ± 8.6	<0.05
Transferrin (md/dL)	260.5 ± 5.5	258.4 ± 6.7	ns
Ferritin (ng/mL)	158.0 ± 3.8	140.5 ± 4.1	<0.02
Albumin (g/dL)	44.2 ± 4.4	41.3 ± 4.0	<0.007

Acronyms: SHG: self-healing group; NIG: needing intervention group; VSD: ventricular septal defect; BSA: body surface area; Hb: haemoglobin; Ht: hematocrit; HbF: fetal haemoglobin. The variables were compared by Student’s *t*-test.

## Data Availability

With the corresponding author. Available on request.
